# Auswirkungen der neuen Mindestmengen in der Viszeralchirurgie auf die Gesundheitsversorgung in Brandenburg aus der Perspektive der Versorger:innen

**DOI:** 10.1007/s00104-023-01971-1

**Published:** 2023-10-26

**Authors:** C. M. Kugler, S. Gretschel, J. Scharfe, S. Pfisterer-Heise, R. Mantke, D. Pieper

**Affiliations:** 1 Fakultät für Gesundheitswissenschaften Brandenburg, Institut für Versorgungs- und Gesundheitssystemforschung (IVGF), Medizinische Hochschule Brandenburg (Theodor Fontane), Immanuel Klinik Rüdersdorf, Seebad 82/83, 15562 Rüdersdorf bei Berlin, Deutschland; 2 Zentrum für Versorgungsforschung Brandenburg (ZVF-BB), Medizinische Hochschule Brandenburg (Theodor Fontane), Rüdersdorf bei Berlin, Deutschland; 3 Klinik für Allgemein‑, Viszeral‑, Thorax- und Gefäßchirurgie, Universitätsklinikum Ruppin-Brandenburg (ukrb), Medizinische Hochschule Brandenburg, Neuruppin, Deutschland; 4 Medizinische Hochschule Brandenburg, Neuruppin, Deutschland; 5 Klinik für Allgemein- und Viszeralchirurgie, Universitätsklinikum Brandenburg an der Havel (ukb), Medizinische Hochschule Brandenburg, Brandenburg an der Havel, Deutschland; 6 Fakultät für Gesundheitswissenschaften Brandenburg, Medizinische Hochschule Brandenburg, Brandenburg an der Havel, Deutschland

**Keywords:** Zentralisierung, Pankreas, Ösophagus, Ländliche Versorgung, Kliniklandschaft, Volume thresholds, Pancreas, Esophagus, Rural healthcare, Centralization

## Abstract

**Hintergrund:**

In der Viszeralchirurgie wurden die Mindestmengen (Mm) für komplexe Eingriffe am Ösophagus und Pankreas 2023 bzw. 2025 angehoben. Kliniken, die die Mm nicht erreichen, dürfen diese Eingriffe nicht mehr durchführen und haben keinen Vergütungsanspruch.

**Ziel der Arbeit:**

Die Studie beantwortet die Fragen, welche Auswirkungen die an der Versorgung im Land Brandenburg Beteiligten durch die neuen Mm erwarten und welche Lösungsansätze sie für das dünn besiedelte Flächenland sehen.

**Material und Methoden:**

Es wurden 19 Expert:inneninterviews mit Krankenhausangestellten (Chefärzt:innen, Oberärzt:innen, Pflegedirektor:innen), niedergelassenen Ärzt:innen und Patient:innenvertretungen im Zeitraum 07/2022 bis 01/2023 durchgeführt. Die Datenauswertung erfolgte inhaltsanalytisch.

**Ergebnisse:**

Die Interviewten erwarteten für die operative Versorgung eine Umverteilung in wenige Kliniken (Zentren); hingegen würden mehr Kliniken, die die komplexen Operationen nicht (mehr) durchführen dürfen, die Funktion von „Portalkliniken“ für die Basisversorgung, Diagnostik und Nachsorge übernehmen. Die Umverteilung könnte auch Auswirkungen auf nicht direkt von der Mm-Regelung betroffene Behandlungen haben. Die Erhöhung der Mm habe außerdem Auswirkungen auf die Weiterbildung und Personalgewinnung. Als Lösungsvorschlag wurden insbesondere Kooperationen zwischen verschiedenen Kliniken diskutiert, die strukturell zunächst gefördert werden müssten.

**Diskussion:**

Mm beeinflussen nicht nur Ergebnisqualität und Erreichbarkeit, sondern ziehen eine Vielzahl weiterer Effekte nach sich. Insbesondere für dünn besiedelte Regionen stellen Mm Herausforderungen für den Zugang zu Ösophagus- und Pankreasoperationen sowie die Kommunikation zwischen Zentren und Portalkliniken bzw. ambulanten Versorger:innen dar.

## Abstract (en)

**Background:**

The legally prescribed minimum volume standards for complex esophageal and pancreatic surgery have been increased or will increase in 2023 and 2025, respectively. Hospitals not reaching the minimum volume standards are no longer allowed to perform these surgeries and are not entitled tor reimbursement.

**Objective:**

The study aims to explore which effects are expected by healthcare professionals and patient representatives and what possible solutions exist for Brandenburg, a rural federal state in northeast Germany.

**Material and methods:**

In this study 19 expert interviews were conducted with hospital employees (head/senior physicians, nursing director), resident physicians and patient representatives between July 2022 and January 2023. The data analysis was based on content analysis.

**Results:**

Healthcare professionals and patient representatives expect a redistribution into a few clinics for surgical care (specialized centres); conversely more clinics that do not (no longer) perform the defined surgeries but could function as gatekeeping hospitals for basic care, diagnostics and follow-up (regional centres). The redistribution could also impact forms of treatment that are not directly defined within the regulation for minimum volume standards. The increased thresholds could also affect medical training and staff recruitment. A solution could be collaborations between different hospitals, which would have to be structurally promoted.

**Conclusion:**

The study showed that minimum volume standards not only influence the quality of outcomes and accessibility but also have a multitude of other effects. Particularly for rural regions, minimum volume standards are challenging for access to esophageal and pancreatic surgery as well as for communication between specialized and regional centres or resident providers.

Mindestmengen wurden als Maßnahme zur Zentralisierung und Sicherstellung einer Mindestqualität eingeführt. In einem dünn besiedelten Flächenland sind Mindestmengen mit besonderen Herausforderungen verbunden.

## Hintergrund und Fragestellung

Die Pankreas- und Ösophaguschirurgie in Deutschland weisen im internationalen Vergleich eine hohe Krankenhaussterblichkeit auf; Komplikationsraten sind mit 27–38 % die höchsten in der Viszeralchirurgie [2]. Für viele Prozeduren besteht ein Zusammenhang zwischen der Fallzahl und der Behandlungsqualität (Volume-Outcome-Beziehung), welcher für die Ösophagus- und Pankreaschirurgie moderat bis stark ist [24]. Beispielsweise hatten Krankenhäuser mit den meisten Ösophagusoperationen in den Jahren 2010 bis 2015 in Deutschland eine um die Hälfte reduzierte Krankenhausmortalität im Vergleich zu Krankenhäusern der geringsten Fallzahl. Dies wird vor allem auf das Rettungsversagen („failure to rescue“) in Krankenhäusern mit kleinen Fallzahlen zurückgeführt, da Komplikationen in Krankenhäusern mit hoher Fallzahl nicht seltener sind, Patient:innen mit Komplikationen dort jedoch seltener versterben [22]. Zudem zeigte sich ein Überlebensvorteil für Patient:innen, die in zertifizierten Krebszentren behandelt wurden [27].

Im Jahr 2004 wurden in Deutschland Mindestmengenregelungen eingeführt. Krankenhäuser müssen jährlich in einem Prognoseverfahren darlegen, dass sie die Mindestmenge im nächsten Jahr erreichen, um einen Vergütungsanspruch zu haben [12]. Der Gemeinsame Bundesausschuss hat die Mindestmenge für komplexe Eingriffe am Organsystem Ösophagus ab 2023 von 10 auf 26 erhöht; für komplexe Eingriffe am Organsystem Pankreas ab 2025 von 10 auf 20 [8, 9]. Insgesamt gab es an 590 Krankenausstandorten 12.327 komplexe Pankreaseingriffe (2019) und an 327 Standorten 3697 komplexe Ösophaguseingriffe (2018; [14, 15]). Laut Simulationen haben die neuen Mindestmengen regional unterschiedlich starke Auswirkungen [14, 15]. Das Bundesland Brandenburg (im Folgenden: Brandenburg) ist mit ca. 2,5 Mio. Menschen vorwiegend dünn besiedelt [7]. Im Jahr 2023 haben von 54 Krankenhäusern in Brandenburg 2 bzw. 14 Krankenhäuser eine Berechtigung zur Leistungserbringung komplexer Ösophagus- bzw. Pankreaseingriffe (Tab. 1; [1]).

**Table 1:** 

Krankenhaus	Berechtigung Ösophagus	Fallzahl Ösophagus 2021	Berechtigung Pankreas	Fallzahl Pankreas 2021	DKG-Zertifizierung
Klinikum Ernst von Bergmann Potsdam	Ja	25	Ja	37	Pankreas, Speiseröhre, viszeral-onkologisches Zentrum
Klinikum Frankfurt (Oder)	Ja (erstmalige Leistungserbringung)		Ja	14	–
Carl-Thiem-Klinikum Cottbus	Nein	–	Ja	56	–
Alexianer St. Josefs-Krankenhaus Potsdam Sanssouci	Nein	–	Ja	8	–
Universitätsklinikum Brandenburg an der Havel	Nein	–	Ja	14	–
KMG Klinikum Luckenwalde	Nein	–	Ja	5	–
Helios Klinikum Bad Saarow	Nein	–	Ja	13	–
Immanuel Klinik Rüdersdorf	Nein	–	Ja	17	–
Klinikum Barnim Werner Forßmann Krankenhaus (Eberswalde)	Nein	–	Ja	5	–
Krankenhaus Märkisch Oderland, Standort Wriezen	Nein	–	Ja	16	–
Asklepios Klinik Uckermark (Schwedt/Oder)	Nein	–	Ja	10	–
Immanuel Klinikum Bernau	Nein	–	Ja	20	–
Universitätsklinikum Ruppin-Brandenburg (Neuruppin)	Nein	–	Ja	25	–
Krankenhaus Märkisch Oderland, Standort Strausberg	Nein	–	Ja (erstmalige Leistungserbringung)		–
**Gesamtanzahl Kliniken mit Berechtigung**	2 Kliniken	–	14 Kliniken	–	–

*DKG* Deutsche Krebsgesellschaft

Diese Studie soll zwei Forschungsfragen untersuchen:

1. Welche Auswirkungen erwarten die an der Versorgung Beteiligten durch die neuen Mindestmengen in der Viszeralchirurgie auf die Versorgung in Brandenburg?
2. Welche Lösungsmöglichkeiten sehen sie zur Gewährleistung einer qualitativ hochwertigen Versorgung bei Zentralisierung?

## Studiendesign und Untersuchungsmethoden

Vorab wurde ein Studienprotokoll registriert [16].

### Stichprobe

Um eine breite Vielfalt der interviewten Personen und ihrer Perspektiven zu erreichen, wurden die Krankenhäuser und niedergelassenen Ärzt:innen zielgerichtet ausgewählt, je nachdem wie sie von der Mindestmenge betroffen waren, nach der geografischen Lage und dem Facharzttitel bzw. der Profession. Unter den 19 Interviewteilnehmenden war die Mehrzahl männlich, in der Chirurgie und im weiteren Metropolraum tätig (Tab. 2). 15 Interviews wurden mit Krankenhausangestellten (Chefärzt:innen, Oberärzt:innen, Pflegedirektor:innen) durchgeführt, die mehrheitlich in Krankenhäusern arbeiteten, die auch schon vor 2022 keine Erlaubnis für Ösophagusoperationen hatten, aber aktuell eine Erlaubnis für Pankreasoperationen haben. Ferner nahm eine Patientenvertreterin aus Brandenburg teil.

**Table 2:** 

Charakteristika	Krankenhaus	Ambulant	Patient:innenvertretung
**Summe**	15	3	1
**Geschlecht**			
Männlich	15	1	0
Weiblich	0	2	1
**Facharzt**			
Chirurgie	8	1	–
Gastroenterologie	3	0	–
Onkologie	2	1	–
Strahlentherapie	1	0	–
Allgemeinmedizin	0	1	–
**Nichtärztliche Berufsgruppen**			
Pflege	1	0	–
**Standort*** (Definition des Amts für Statistik Berlin-Brandenburg)*			
Berliner Umland	4	0	–
Weiterer Metropolraum	11	3	–
**Betroffenheit durch Mindestmenge für komplexe Ösophaguseingriffe**			
Unterschied: Erlaubnis 2022, aber nicht nach der neuen Regelung	2	–	–
Unverändert: Erlaubnis 2022 und nach neuer Regelung	1	–	–
Unverändert: keine Erlaubnis 2022 und danach	12	–	–
**Betroffenheit durch Mindestmenge für komplexe Pankreaseingriffe**			
Unterschied: Erlaubnis 2022, aber nicht nach der neuen Regelung^a^	8	–	–
Unverändert: Erlaubnis 2022 und nach neuer Regelung^a^	5	–	–
Unverändert: keine Erlaubnis 2022 und danach	2	–	–

^a^Basierend auf Fallzahlen 2021–2022

### Ablauf

Ein semistrukturierter Leitfaden wurde für die Durchführung der Interviews erarbeitet und pilotiert. Die Interviews wurden je nach Präferenz der Teilnehmenden telefonisch, per Videokonferenz oder in Präsenz von zwei Versorgungsforscherinnen (CMK, SPH) durchgeführt, aufgezeichnet, transkribiert und inhaltsanalytisch mit MAXQDA 2022 (Version 22.2.0, VERBI GmbH, Berlin, Deutschland) ausgewertet (CMK, JS; [20]). Im Mittel dauerten die Interviews 31 min (Spanne: 13–46). Es wurde eine Datensättigung erreicht, d. h., dass in den letzten drei Interviews keine neuen Themen auftauchten.

## Ergebnisse

Die Inhalte der Expert:inneninterviews lassen sich in drei Themenbereiche einteilen:

- Auswirkungen der neuen Mindestmengen,
- Lösungsansätze und
- Akzeptanz von Zentralisierung.

### Auswirkungen der neuen Mindestmengen

Die Interviewten erwarteten, dass durch die neuen Mindestmengen nur wenige Zentren (für Ösophagusoperationen nur ein Zentrum) mit steigenden Fallzahlen für Ösophagus- und Pankreasoperationen bestehen bleiben. Zugleich würden vermehrt „Portalkliniken“ entstehen, die die Basisversorgung, Diagnostik und Nachsorge übernehmen können, aber selbst keine Pankreas- und Ösophagusoperationen mehr anbieten. Die fehlende Vernetzung zwischen Zentren und „Portalkliniken“ bzw. ambulanten Versorger:innen könne die Zusammenarbeit in der Behandlung von Patient:innen und den interdisziplinären Austausch erschweren. Zugleich könnten verbleibende Zentren überflutet werden, was zu langen Wartezeiten oder zur Selektion von Patient:innen führen könne.

Mindestmengen könnten sich auch auf andere, nicht direkt von der Richtlinie betroffene Behandlungen auswirken, z. B. Karzinome, die in das Pankreas infiltrieren, die Thoraxchirurgie, wo man „nicht mehr so zu Hause“ sei, wenn man keine Ösophaguschirurgie mehr anbietet. Auch könne sich die Versorgung postoperativer Komplikationen nach Entlassung bzw. von (seltenen) Notfällen durch den Erfahrungsverlust in den „Portalkliniken“ verschlechtern. In den „Portalkliniken“ wurde eine qualitativ gute Diagnostik und Nachsorge infrage gestellt, z. B. weil keine Endoskopiker:innen vor Ort mehr beschäftigt seien.

Die Mindestmengen bieten nach Aussagen der Behandler:innen Fehlanreize für Fehlkodierungen und Fehlversorgung bzw. Indikations- und Mengenausweitungen. Interessanterweise sahen Interviewte die Möglichkeit der Indikationsausweitung bei anderen, aber nicht bei sich selbst: „Also ganz klar, die Gefahr besteht (…) Wenn Sie 26 haben müssen, und Sie haben im Oktober meinetwegen 22 aus eigener Kraft, dann ist natürlich die Verleitung nah, auf 27 zu kommen. (…) Also, ich kann das für mich ausschließen, ja, aber dass es nicht passiert, kann man definitiv nicht ausschließen“ (Chirurg, weitere Metropolregion).

Betroffene Patient:innen könnten einerseits von einer Verbesserung der operativen Behandlungsqualität profitieren, wenn sie in Zentren mit größeren Fallzahlen operiert werden. Auf der anderen Seite müssten sie komplexere Versorgungswege (mehr involvierte Akteur:innen) und zum Teil weite Anfahrten befürchten. Weite Anfahrten seien je nach Generation und Wohnort akzeptabel oder nicht – zumeist ließen sich Patient:innen von der Behandlung in einem weiter entfernten Zentrum überzeugen. Auch Besuche durch Angehörige würden durch eine längere Anfahrt erschwert.

Ein wichtiges Thema war die Auswirkung auf die ärztliche Weiterbildung (Facharzttitel für Chirurgie, Weiterbildung Spezielle Viszeralchirurgie). Diese Einschränkungen führten aus Sicht der Interviewten auch zu einer erschwerten Personalgewinnung, weil es für Ärzt:innen attraktiver sei, in Zentren zu arbeiten, in denen sie die gesamte Weiterbildung abschließen können. Dies könne bereits bestehende Personalengpässe vergrößern und bei weiteren Mindestmengen zu einer Zersplitterung der Viszeralchirurgie führen.

**Figure 1:**
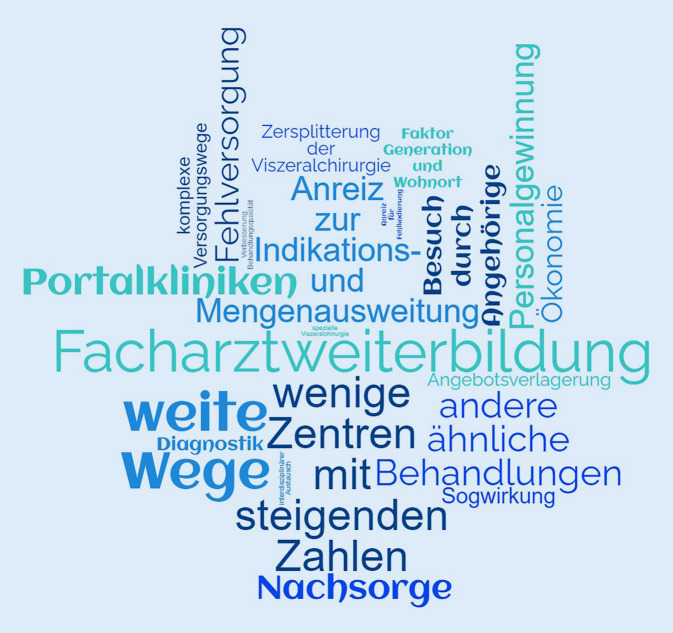

Die Mindestmengen hätten auch ökonomische Auswirkungen, weil große, hochpreisige Operationen in mittelgroßen Kliniken wegfallen: „Mit einer Hernienoperation (…) kann man keine Klinik führen. Wir brauchen natürlich auch die großen Eingriffe und ich meine, das sind ja auch die Eingriffe, die sozusagen das Geld in die Kasse bringen“ (Chirurg, weitere Metropolregion). Diese ökonomischen Folgen könnten Angebotsverlagerungen bewirken. Mindestmengen könnten auch zu Sogwirkungen führen in dem Sinne, dass sich der Ruf von Krankenhäusern bei Zuweiser:innen verändert und diese dann nicht mehr in die „Portalkliniken“ einweisen. Abb. 1 stellt die diskutierten Themen als Wortwolke dar.

### Lösungsansätze

Eine Option für die Versorgung von Ösophagus- bzw. Pankreaspatient:innen aus Brandenburg bei den höheren Mindestmengen sei es, diese Patient:innen nach Berlin oder andere große Städte zu verweisen, was aufgrund der demografischen Struktur in Brandenburg auch kritisch gesehen wurde.

Als Lösungsansatz wurden vor allem Kooperationen zwischen Kliniken diskutiert: Regionale Netzwerke sollten nach Ansichten der Interviewten vermeiden, dass mehrere Kliniken die Mindestmengen knapp nicht erreichen, um so sicherzustellen, dass weiterhin Kliniken in Brandenburg Pankreaseingriffe anbieten: „Ich meine, (…) wir müssen natürlich gucken als Brandenburger Haus, dass wir diese Eingriffe auch in Brandenburg behalten, weil sonst haben wir insgesamt in Brandenburg eine Wüste, wo wir diese Eingriffe gar nicht mehr machen können“ (Chirurg, weitere Metropolregion). Damit sich gute Kooperationen etablieren können, müssten diese strukturell gefördert werden. Gute Kooperationen könnten die ärztliche Weiterbildung innerhalb von Rotationsplänen ermöglichen. Zugleich könnte durch die Zentren und ihre Netzwerke die Zusammenarbeit mit Patient:innenvertretungen verbessert werden.

Als weitere Lösungsansätze wurden Dienstleistungen und die Digitalisierung diskutiert, die aber auch kritisch gesehen wurden.

### Akzeptanz von Zentralisierung bzw. Mindestmengen

Für eine Zentralisierung sprachen aus Sicht der Interviewten Studien, die eine Volume-Outcome-Beziehung nachwiesen. Bei Krankenhäusern mit höherer Fallzahl sei ein besseres Komplikationsmanagement zu erwarten, insbesondere in der Ösophaguschirurgie. Zudem würden für diese Operationen eine gewisse Erfahrung, interdisziplinäre Behandlung und Strukturqualität benötigt. Auf der anderen Seite wurden Mindestmengen kritisch gesehen, z. B. gab es unter den Interviewten keine Akzeptanz für die Mindestmenge von 26 Ösophaguseingriffen, weil sie keine „runde“ Zahl wie etwa 20 oder 25 ist. Die Zahl erschien willkürlich und einige vermuteten eine verborgene Strategie, bei der so lange erhöht wurde, bis genügend Krankenhäuser ausgeschlossen werden. Den Befürworter:innen der Mindestmengen wurde unterstellt, dass sie von ihnen profitieren würden. Ein (bevorstehender) Ausschluss von der Versorgung schien verbunden zu sein mit Emotionalität und dem Gefühl, etwas weggenommen zu bekommen. Die Interviewten schlugen alternative Maßnahmen für eine Zentralisierung vor: Zentralisierung solle sich an Spezialisierungsgruppen anstatt an einzelnen Organen orientieren; Struktur- und Prozessqualität sollten eine größere Rolle spielen als einfache Zahlen. Interviewte wünschten sich eine grundlegende gesundheitspolitische Planung anstatt der Vorgabe von Zahlen.

## Diskussion

Die Studie beleuchtete die Perspektive der an der Versorgung von Ösophagus- und Pankreaspatient:innen Beteiligten aus Brandenburg. Mindestmengen können neben der Verbesserung der operativen Outcomes bei gleichzeitig längerer Anfahrt viele weitere Effekte haben. Insbesondere könnte sich in der Fläche die Gesamtversorgungsqualität (Diagnostik, Komplikationen) verschlechtern und die ärztliche Weiterbildung erschwert werden. Dies können Volume-Outcome-Studien nicht beantworten; hierfür werden Studien benötigt, die eine Mindestmengenregelung als Intervention betrachten – und die sind rar.

### Verbesserungen durch Zentralisierung

In anderen Ländern konnte Zentralisierung die Krankenhausmortalität reduzieren: In England wurde die Behandlung von Ösophagus-Magen-Karzinomen von 113 Zentren auf 34 Zentren zentralisiert, sodass die mediane Fallzahl in den Zentren von 21 auf 55 stieg. In derselben Zeit sank die 30-Tages-Mortalität von 7,4 auf 2,5 % [30]. In den bez. Zentralisierung der Pankreaschirurgie untersuchten Regionen in Kanada (Ontario mit Zentralisierungsstrategie, Quebec ohne Zentralisierungsstrategie) stieg der Anteil der in Zentren operierten Patient:innen an, aber nur in Ontario sank im gleichen Zeitraum die 30-Tages-Mortalitätsrate [28].

### „Portalklinik“-Konzept

Interviewte antizipierten, genau wie Lang und Kollegen, dass sich die Gesamtversorgungsqualität im „Portalklinik“-Konzept verschlechtern könnte [18]. Nach der Zentralisierung von Krebszentren in England berichtete das Personal, dass die Erfahrung in lokalen Zentren für eine angemessene Diagnostik und Versorgung von Komplikationen nicht ausreichte [5]. Patient:innen, die nach einer Pankreatektomie in dasselbe Krankenhaus wiedereingewiesen wurden, hatten eine geringere 90-Tages-Mortalität, als diejenigen, die in ein anderes Krankenhaus eingewiesen wurden [6]. Bei der adjuvanten Chemotherapie des Pankreaskarzinoms war es hingegen unerheblich, ob diese in derselben Institution wie die Operation oder in einer anderen stattfand [19].

### Weiterbildung

Durch die Mindestmengen wird die ärztlich-chirurgische Weiterbildung deutlich erschwert [18, 23]. Die Weiterbildung der speziellen Viszeralchirurgie könnte den europäischen Anforderungen angeglichen werden [23]. In dieser Studie wurde, wie bereits an anderer Stelle, eine Weiterbildung innerhalb von Rotationsplänen in regionalen Netzwerken vorgeschlagen [18, 23, 26]. Diese müssten jedoch zunächst etabliert werden. Rotationspläne müssten der Gefahr einer Verlängerung der Weiterbildungszeit begegnen sowie Anreize für Zentren und das Personal schaffen diese Möglichkeit anzunehmen [26].

### Fahrtzeiten und Angehörigenbesuche

Nach Simulationen verlängern sich die Fahrtzeiten durch die erhöhten Mindestmengen zu Kliniken für Ösophagusoperationen im deutschlandweiten Durchschnitt nur um 11 min (insgesamt 31 min), für Pankreasoperationen um 3 min (insgesamt 21 min), jedoch benötigen 5 % der Patient:innen mehr als 63 bzw. 51 min [14, 15]. Allerdings fahren Patient:innen im Durchschnitt bereits 22–24 min weiter als nötig und „überspringen“ das nächste versorgende Krankenhaus [3]. Eine weite Entfernung zur Behandlung kann bei Krebspatient:innen mit einem reduzierten Überleben zusammenhängen [29]. Gleichzeitig kann der Besuch durch Angehörige erschwert sein, der für die psychologische Unterstützung operierter Personen wichtig ist [13].

### Strategien zur Zentralisierung

Die Orientierung an einfachen Zahlen löste Kritik aus [17, 18]. Deutschland könnte sich an England orientieren und die Zentralisierung mit einer gesundheitspolitischen Planung und einem Implementationsprozess mit regionalen Netzwerken einführen anstatt einer „Selbstregulation des Marktes durch Mindestmengen“ [5, 11]. Durch eine Simulation der Patient:innenströme je Krankenhaus könnten verbleibende Pankreas- und Ösophaguschirurgiekliniken ihre Kapazitäten anpassen, um eine befürchtete Verlängerung der Wartezeit zu vermeiden. Die Pläne zur Krankenhausreform beinhalten zum Zeitpunkt der Manuskripteinreichung keine Mindestmengen mehr, sondern Leistungsgruppen, angelehnt an die Krankenhausplanung in Nordrhein-Westphalen. Dort ist im Leistungsbereich „Viszeralchirurgie“ jeweils eine Leistungsgruppe für Ösophagus- und Pankreaseingriffe vorgesehen [21]. Weil Erkrankungen nicht an Organgrenzen aufhören, wäre eine Aufteilung in oberer/unterer Gastrointestinaltrakt, hepatobiliopankreatische/endokrine Chirurgie, wie oftmals üblich, empfehlenswert [4]. Dies wird unterstützt durch das Ergebnis, dass die Mortalität in Krankenhäusern mit kleiner Fallzahl an Pankreaskopfresektionen, aber hoher Fallzahl pankreasnaher Operationen ähnlich gut war wie in Krankenhäusern mit hoher Fallzahl [10]. Eine strukturelle Förderung von Kooperationen für Versorsungswegen von Patient:innen würde dem „Bratislava Statement: consensus recommendations for improving pancreatic cancer care“ entsprechen, das empfiehlt, Netzwerke zu stärken – gerade weil nur wenige Pankreaskarzinompatient:innen operiert werden [25].

### Limitationen

Die Studie hat einige Limitationen. 1. Es wurden Erwartungen über die Auswirkungen der Mindestmengen untersucht, die nicht mit den tatsächlichen Auswirkungen übereinstimmen müssen. Viele deckten sich jedoch mit retrospektiven Erfahrungen aus England [5]. 2. Trotz des umfassenden und zielgerichteten Stichprobendesigns wurden keine Assistenzärzt:innen oder Studierende interviewt. 3. Alle Klinikangestellten waren männlich, weil Frauen in den entsprechenden Positionen fehlten. 4. Es wurde nur eine Patient:innenvertreterin interviewt, weil in Brandenburg kaum Patient:innenvertretungen aufzufinden waren. 5. Es wurde die Perspektive aus Berlin bez. Spekulationen über die Überflutung der Zentren und verlängerten Wartezeiten nicht untersucht. Unter den Interviewten war jedoch auch eine Person an einem großen Krebszentrum in Brandenburg.

## Fazit für die Praxis

- Für die Zentralisierung wird eine Implementationsstrategie anstatt einer „Selbstregulation des Marktes durch Mindestmengen“ benötigt. Dies beinhaltet die strukturelle Förderung von Kooperationen für Versorgungswege von Patient:innen und die Weiterbildung.
- In Brandenburg wird eine politische Strategie benötigt, die Versorgungsstrukturen aktiv mitzugestalten, um wenige, regional verteilte Kliniken für die Pankreaschirurgie zu bestimmen. So könnte verhindert werden, dass ein Großteil der aktuell 14 Krankenhäuser knapp die Leistungsmenge für Pankreaseingriffe verfehlt. Hier ist schnelles Handeln gefordert, weil für die Prognose ab 2025 die Fallzahlen im Jahr 2023/24 entscheidend sind. Auch kann die Möglichkeit der Ausnahmeregelung erwogen werden.
